# Phylogenetics of *Mycoplasma hominis* clinical strains associated with gynecological infections or infertility as disclosed by an expanded multilocus sequence typing scheme

**DOI:** 10.1038/s41598-018-33260-x

**Published:** 2018-10-05

**Authors:** Safa Boujemaa, Amina Ben Allaya, Béhija Mlik, Helmi Mardassi, Boutheina Ben Abdelmoumen Mardassi

**Affiliations:** 1 Group of Mycoplasmas, Laboratory of Molecular Microbiology, Vaccinology, and Biotechnology Development, Institut Pasteur de Tunis, Université de Tunis El Manar, 13, Place Pasteur, BP 74, 1002 Tunis Belvédère, Tunisia; 2 Unit of Typing & Genetics of Mycobacteria, Laboratory of Molecular Microbiology, Vaccinology, and Biotechnology Development, Institut Pasteur de Tunis, Université de Tunis El Manar, 13, Place Pasteur, BP 74, 1002 Tunis Belvédère, Tunisia

## Abstract

To our knowledge, the phylodistribution of *M. hominis* clinical strains associated with various pathological conditions of the urogenital tract has not been explored hitherto. Here we analyzed the genetic diversity and phylogenetic relationships among 59* M. hominis* Tunisian clinical isolates, categorized as gynecological infections- or infertility-associated pathotypes. For this purpose, we developed an expanded multilocus sequence typing (eMLST) scheme, combining the previously reported multilocus sequence typing (MLST) loci (*gyrB, tuf, ftsY, uvrA, gap*) with a new selected set of putative virulence genes (*p120’, vaa, lmp1, lmp3, p60*), referred herein to as multi-virulence-locus sequence typing (MVLST) loci. In doing so, *M. hominis* population was segregated into two distinct genetic lineages, which were differentially associated with each pathotype. Such a clear dichotomy was supported by several phylogenetic and population genetic analysis tools. Recombination was found to take place, but not sufficient enough to break down the overall clonal population structure of *M. hominis*, most likely as a result of purifying selection, which accommodated the most fit clones. In sum, and owing to the eMLST scheme described herein, we provide insightful data on the phylogenetics of *M. hominis*, arguing for the existence of genetically differentiable urogenital pathotypes.

## Introduction

*Mycoplasma hominis*, which belongs to the *Mycoplasmataceae* family, in the *Mollicutes* class, was the first mycoplasma species isolated from humans in 1937^1^. It resides, as a commensal, in the lower urogenital tract of healthy persons. Under certain circumstances, *M. hominis* can cause a variety of genital infections such as bacterial vaginosis, pelvic inflammatory disease, and cervicitis^2^. This microorganism seems to be associated with pregnancy complications and neonatal diseases^3^. In addition, several studies reported the pathogenic role of *M. hominis* in infertility^4,5^. More interestingly, this species has been linked to a wide range of extragenital infections (septic arthritis, endocarditis, brain abscess), especially in immunocompromised patients^6–8^.

To better understand the epidemiology and the mode of spread of *M. hominis*, several molecular typing systems have been developed. These include Pulse-Field Gel Electrophoresis (PFGE), Restriction Fragment Length Polymorphism (RFLP) analysis, Amplified Fragment Length Polymorphism (AFLP), and Random Amplified Polymorphic DNA (RADP). All these methods have revealed a high degree of both genetic and antigenic heterogeneity among *M. hominis* strains^9–12^. Although informative, these approaches proved to be quite difficult to reproduce and standardize between laboratories^13^. Recently, a molecular typing method based on Multiple-Locus Variable-Number Tandem Repeat Analysis (MLVA) has been developed^14^. This method proved discriminatory enough to be used for large epidemiological studies, and could also be useful to address transmission dynamics of *M. hominis* at the individual level. However, there was no obvious association between the MLVA type and the isolate’s characteristics^14^. Multilocus Sequence Typing (MLST) assay, targeting housekeeping genes, was found faster and easier to perform, and it exhibited a higher discriminatory power than did other subtyping methods^15^. It was first proposed in 1998 for the characterization of isolates of the human pathogen *Neisseria meningitidis*^16^, and has subsequently been applied to the epidemiologic studies of several bacterial species including mycoplasmas, such as *Mycoplasma agalactiae*, *Mycoplasma bovis*, *Mycoplasma pneumoniae*, and *Mycoplasma synoviae*^17–19^. MLST results are unambiguous, highly reproducible, and portable; and hence, they can be easily comparable between laboratories via worldwide web databases, including MLST (http://www.mlst.net/) and PubMLST (http://pubmlst.org/). An MLST approach was also developed for *M. hominis*, and was mainly used to evaluate the frequency of recombination events rather than to investigate the evolutionary relationships between strains^20^. More recently, Jironkin *et al*., based on the genome sequence of 18 *M. hominis* isolates, has identified three sets of seven genes that could be used as minimum multilocus sequence typing schemas^21^.

However, as far as could be ascertained, none of the existing typing approaches has shown the potential to reliably categorize *M. hominis* isolates according to their involvement in particular pathological conditions. Towards this purpose, we sought here to develop an expanded multilocus sequence typing (eMLST) scheme, which included both housekeeping genes and a set of putative virulence loci, and to assess its ability to distinguish between *M. hominis* clinical strains associated with gynecological infections or infertility.

## Results

### Allelic polymorphism of housekeeping genes

The number of polymorphic sites varied from 2 (*gyrB*) to 36 (*uvrA*), resulting in an allelic variation of 3 (*gyrB*) to 11 (*uvrA* and *tuf*) (Table 1). The haplotype diversity (H) varied from 0.188 (*gyrB*) to 0.840 (*gap*) and the ratio *d*_*N*_/*d*_*S*_ ranged from 0 (for *gyrB and ftsY*) to 0.0798 (for *gap*). The Tajima’s *D* values ranged from −0.267 (*tuf*) to −0.737 (*gap*) and showed no statistical significance, a finding that might be interpreted as the result of purifying selection (Table 1).

**Table 1:** Nucleotide diversity of the 10 eMLST loci.

Gene	Amplicon size (bp)	No. of alleles	No. of polymorphic sites (%)	Haplotype diversity (H)	*d*_*N*_ */d*_*S*_	Tajima’s *D*^a^
*uvrA*	573	11	36 (6.28)	0.839	0.0374	−0.276
*gyrB*	402	3	2 (0.49)	0.188	0	N/A
*ftsY*	435	6	13 (2.99)	0.381	0	−0.458
*tuf*	381	11	10 (2.62)	0.833	0.0328	−0.267
*gap*	570	10	28 (4.91)	0.840	0.0798	−0.737
*p120′*	510	13	19 (3.72)	0.862	0.1739	−0.346
*vaa*	297	6	14 (4.71)	0.700	0.2558	−0.019
*lmp1*	249	18	23 (9.23)	0.907	0.2570	−0.632
*lmp3*	375	13	27 (7.18)	0.885	0.2741	−0.625
*p60*	297	10	13 (4.37)	0.808	0.0843	−0.435

N/A, not available value because there were few sequences to be used in the Tajima’s *D* test; ^a^None of the Tajima’s *D* values significantly deviated from zero (*P* > 0.10).

### Allelic polymorphism of virulence genes

The data reporting the allelic variations are summarized in Table 1. The discriminatory ability of the different loci, measured as number of alleles, varied from 6 (*vaa*) to 18 (*lmp1*). The H value ranged from 0.7 (*vaa*) to 0.907 (*lmp1*), and the ratio *d*_*N*_/*d*_*S*_ varied from 0.0843 (for *p60*) to 0.2741 (for *lmp3*), which suggests that the virulence genes evolved under purifying selection. The Tajima’s *D* statistic varied from −0.019 (*vaa*) to −0.632 (*lmp1*).

### Defining ST, VT, eST, and their distribution within clonal complexes

**Figure 1:**
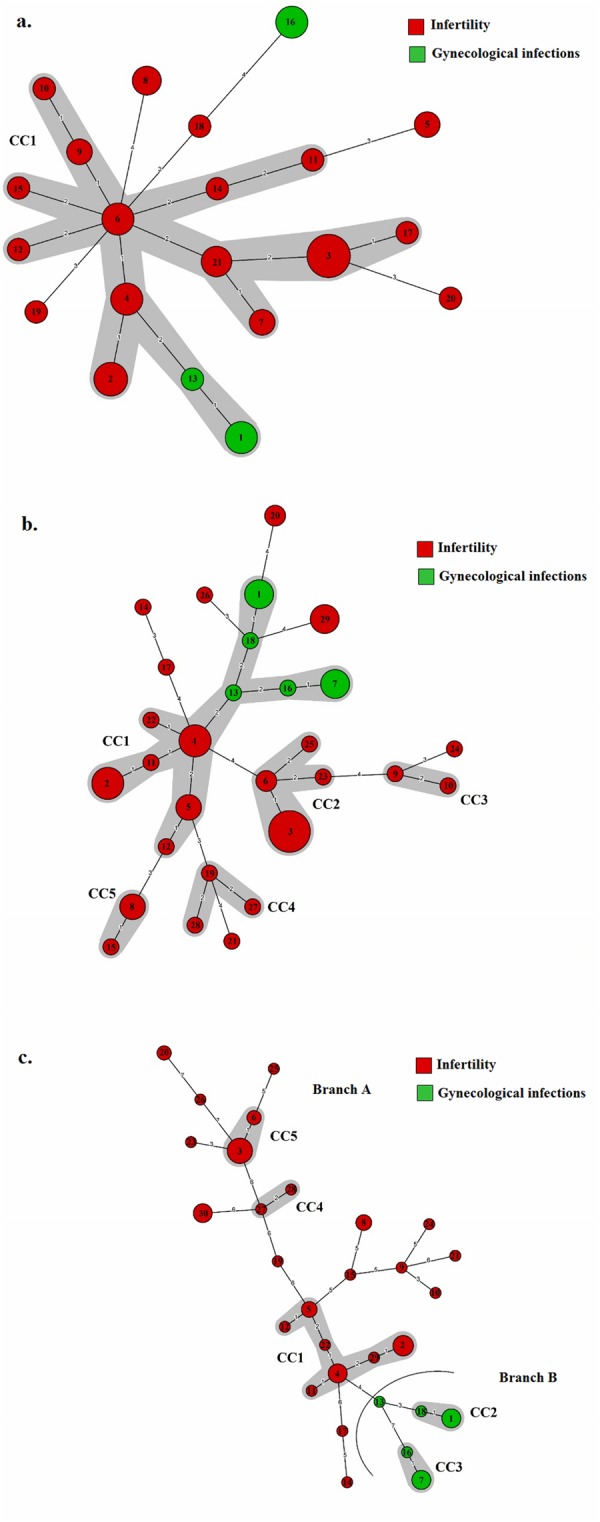
Minimum spanning trees analysis of the 59 *M. hominis* isolates based on MLST (**a**), MVLST (**b**), and eMLST (**c**) data. Each circle corresponds to a distinct allelic profile, and the circle size corresponds to the number of isolates sharing the same profile. The circle was coded by assigning the same color to identical type of clinical manifestations. The shaded zones between certain groups of circles indicate that these profiles belong to the same CC. Numerals connecting the circles indicate the number of allelic differences between the profiles.

The 59 *M. hominis* isolates evaluated by MLST were assigned to 21 STs (Supplementary Table S1), ten of which were represented by a single isolate. Among STs shared by several isolates, the most frequently encountered were ST3 (11 isolates) and ST2 (6 isolates). The eBURST analysis found one clonal complex (CC1) and six singletons (Fig. 1a). The MVLST scheme distinguished 29 VTs (Supplementary Table S1), the majority of which (19 of 29 VTs) consisted of a single isolate. The most frequently VTs were VT3 (9 isolates), VT4, and VT2 (5 isolates). eBURST analysis defined one major CC (CC1), four minor CCs (CC2 to CC5), and seven singletons. The main clonal complex CC1 included 26 strains (44% of the total) that were resolved into 11 VTs. Of these, 5 and 6 VTs were associated to gynecological infections and infertility, respectively. The remaining CCs and singletons were linked to infertility (Fig. 1b). eMLST analysis resulted in the delineation of 30 eSTs (Supplementary Table S1). Twenty of the eSTs (66.67%) were represented by a single isolate. The most frequently eSTs were eST3 (9 isolates), followed by eST2 (5 isolates) eST4, eST7, and eST30 (4 isolates). eBURST resolved the 30 eSTs into one major CC (CC1), four minor CCs (CC2 to CC5), and 15 singletons. The main clonal complex CC1 contained 7 eSTs, including 15 strains (25.42% of the total). Within this CC, eST4 was the predicted ancestral type. Interestingly, the minimum spanning tree (MST) generated from eMLST data showed two separate branches: branch A which grouped all eSTs related to infertility (CC1, CC4, CC5, and 14 singletons), and branch B which grouped all eSTs related to gynecological infections (CC2, CC3, and eST13) (Fig. 1c).

### Evaluating the discriminatory power of eMLST

The Simpson’s index of diversity (SID) of eMLST was 0.956, which is an ideal cutoff value for a molecular typing method^22^. Of note, the SID of MVLST and MLST gene subsets yielded slightly lower values (0.954 and 0.932, respectively), thus justifying their combination in an eMLST scheme. The SID of the MVLST gene subset was very close to that of eMLST, suggesting its possible use at first intention, inasmuch as the highest overall congruence, as assessed by the adjusted Rand index (AR), was found between eMLST and MVLST (97.4%) (Table 2). However, based on the adjusted Wallace coefficient (AW), the eMLST scheme developed herein proved to be the most powerful and adequate to type *M. hominis* strains. Indeed, a maximal value of AW was obtained with the 10 eMLST loci combination compared to the MLST and MVLST gene subsets (Table 2).

**Table 2:** Simpson’s Index of Diversity (SID), Adjusted Rand index (AR), and Adjusted Wallace coefficient (AW) of the three typing schemes.

Typing Methods	Simpson’s index of diversity (SID) (95% CI)	Adjusted Rand index (AR) (95% CI)			Adjusted Wallace coefficient (AW) (95% CI)		
		MLST	MVLST	eMLST	MLST	MVLST	eMLST
MLST	0.932 (0.904–0.960)	—	—	—	—	0.633 (0.459–0.807)	0.634 (0.460–0.807)
MVLST	0.954 (0.931–0.977)	0.759 (0.599–0.930)	—	—	0.948 (0.886–1.000)	—	0.949 (0.888–1.000)
eMLST	0.956 (0.933–0.979)	0.776 (0.626–0.937)	0.974 (0.916–1.000)	—	1.000 (1.000–1.000)	1.000 (1.000–1.000)	—

### Phylogenetic analyses

**Figure 2:**
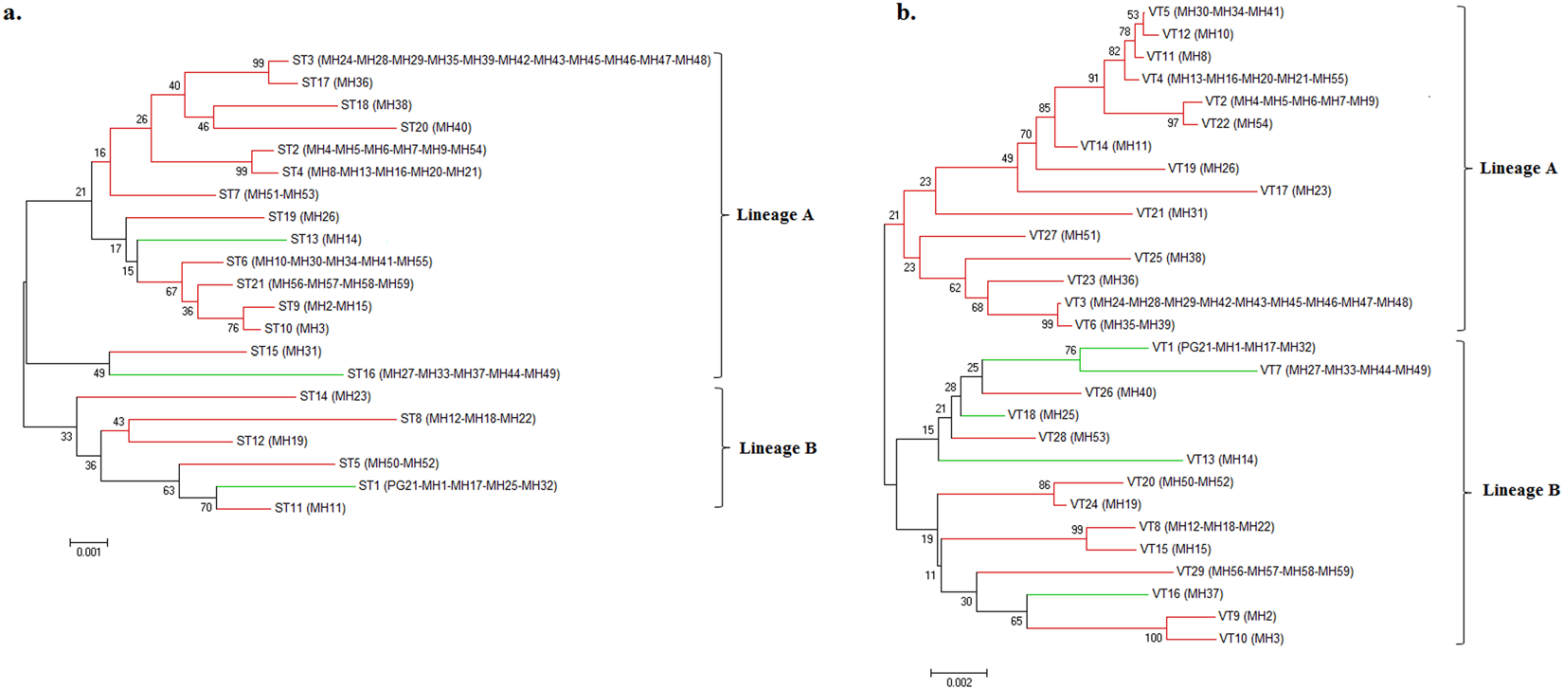
Neighbor-joining trees of 59 *M. hominis* isolates based on the concatenated sequences of MLST (**a**) and MVLST (**b**) data. Bootstrap values (1,000 replications) are shown at the interior branches. The branches are colored according to the association of STs and VTs with infertility (red) or gynecological infections (green).

**Figure 3:**
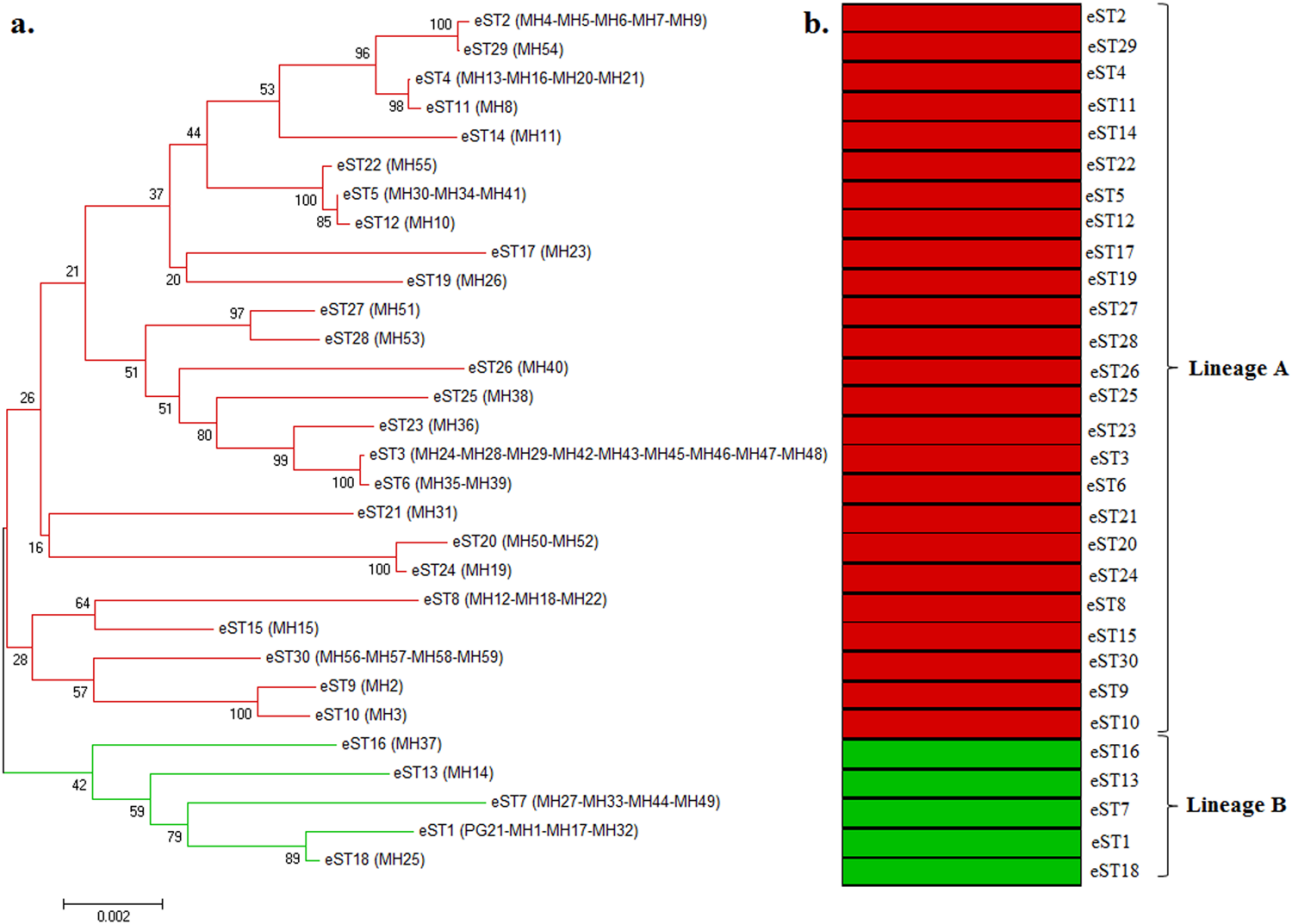
Neighbor-joining (NJ) tree and ancestry of 59 *M. hominis* isolates. (**a**) Phylogenetic NJ tree obtained from the concatenated sequences from the 30 eSTs. Bootstrap values are indicated for all branches. The branches are colored according to the association of eST with infertility (red) or gynecological infections (green). (**b**) Ancestral subpopulations obtained by STRUCTURE analysis. Each bar (stacked vertically) represents 1 of 30 eSTs, ordered on the y-axis by their positions in the NJ tree. The two subpopulations are colored in red (lineage A) and green (lineage B), respectively.

To infer the evolutionary relationships amongst *M. hominis* isolates, three phylogenetic trees based on the concatenated gene sequences from the MLST, MVLST, and eMLST data, were obtained using the Neighbor-joining (NJ) method. For the three schemes, two major lineages, A and B, were distinguished, yet not supported by the bootstrap analysis. The dendrogram generated from MLST data showed that the majority of STs were grouped into lineage A (15 STs), and it also revealed that infertility-associated STs did not constitute a distinct lineage from the gynecological infections-associated ones (Fig. 2a). Whereas, the dendrogram based on the concatenated sequences from the MVLST gene set, revealed an equal distribution of VTs between the two lineages. Specifically, lineage A was composed of strains associated only with infertility, while lineage B contained both types of strains (Fig. 2b). Strikingly, the NJ tree based on eMLST data revealed a convincing association between clinical manifestations and a particular lineage; all infertility-associated strains were clustered into lineage A, while all gynecological-associated strains were clustered into lineage B (Fig. 3a). This result was largely in agreement with the MST topology, which demonstrated two branches: branch A corresponding to lineage A, and branch B corresponding to lineage B (Fig. 1c). Analysis of the phylodistribution of eMLST loci variants showed that *lmp3*, *vaa*, and particularly *tuf* gene alleles, evolved towards a tight association with a specific clinical manifestation (Supplementary Fig. S1). However, none of the MLST-, MVLST-, or eMLST-derived data could be correlated with the patient’s sex or the year of isolation of the clinical strains.

In order to gain deeper insights into the phylodistribution of *M. hominis* clinical strains, a linkage model of STRUCTURE software was applied to the sequence data set of the 30 eSTs. Ten runs with K values from 2 to 10 showed maximal posterior probability at K = 2, indicating that the 30 *M. hominis* eSTs could be subdivided into 2 ancestral populations or lineages (Fig. 3b). Lineage A (red) contained all infertility-associated eSTs (25 eSTs), and lineage B (green) comprised all gynecological infections-associated eSTs (5 eSTs). No admixture of ancestral sources was found between these two populations, suggesting a high homogeneity of the eSTs in each population. Interestingly, the two populations identified from STRUCTURE analysis were in total accordance with the two lineages revealed by NJ tree (Fig. 3a). These results were also consistent with the two branches revealed by the MST (Fig. 1c). The *F*_ST_ value of 0,323 (*P* = 0) further confirmed the high level of genetic differentiation between the two lineages.

Taken together, the above-stated data suggested that *M. hominis* clinical strains associated with gynecological infections or infertility, in Tunisia, are genetically differentiable.

### Recombination in *M. hominis*

**Figure 4:**
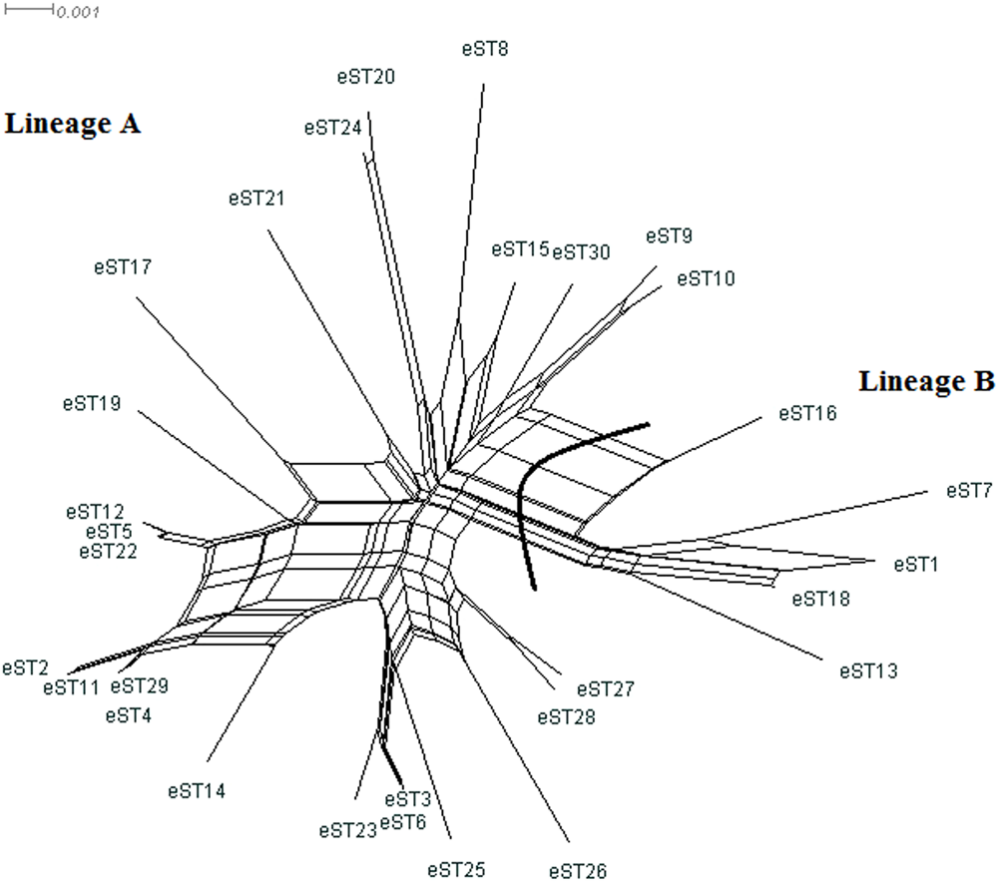
Split network analysis based on the concatenated sequences of 30 eSTs identified among 59 *M. hominis* isolates by neighbor-net method.

A split decomposition analysis, searching evidence for recombination among the ten genes, revealed different structures in the split graphs (Supplementary Fig. S2). The split graphs for *uvrA*, *gap*, and *p60* were network-like with parallelogram structures indicative of recombination, which was statistically confirmed by the *phi*-test (*P* < 0.05). By contrast, no recombination could be detected for *tuf*, *lmp1, lmp3, ftsY*, *p120’*, and *vaa*, whose *phi-*test *P* values were >0.05. For *gyrB*, there were too few informative characters to be used in the *phi*-test. However, strong evidence for recombination (*phi*-test *P* value < 0.05) was observed within and across lineages when the concatenated sequences of eSTs of the entire population were used (Table 3). Indeed, the split decomposition analysis of the 30 eSTs displayed network-like structure with rays of different length and parallelogram-shaped groupings, confirming that recombination events had occurred frequently during *M. hominis* evolution (Fig. 4). There were two distinct clusters in the split tree, which was consistent with the NJ-derived tree and STRUCTURE analysis. Simultaneously, the per site recombination/mutation ratio (ρ/θ) values for all population and individual lineages were calculated in order to estimate the relative contributions of both recombination and mutation to the evolution of *M. hominis* (Table 3). This ratio was below 1 for the whole population of *M. hominis* and for lineage A (0.05166 and 0.005542, respectively), indicating that recombination occurred much less frequently than mutation. By contrast, recombination seems to be a major driving evolutionary force in lineage B, since it was found to occur almost 8 times more likely than mutation.

**Table 3:** Results of recombination test.

Population (n)	*phi-*test	Recombination			Linkage disequilibrium
		ρ/site	θ/site	ρ/θ	*I* ^*S*^_*A*_
Total (30 eSTs)	*P* = 0	6.613 × 10^−3^	0.1279947	0.05166	0.3103 (*P* < 0.001)
Lineage A (25 eSTs)	*P* = 0	7.284 × 10^−4^	0.1341623	0.005542	0.3263 (*P* < 0.001)
Lineage B (5 eSTs)	*P* = 1.095E-6	6.954 × 10^−2^	8.806 × 10^−3^	7.897	0.7171 (*P* < 0.001)

To further assess the likelihood of recombination, we carried out a linkage disequilibrium analysis. The standardized index of association (*I*_*A*_^*S*^) of 0.3103 (*P* < 0.001) for the whole *M. hominis* population indicated a tendency of linkage disequilibrium between the alleles, a finding minimizing the role of recombination (Table 3). Moreover, *I*_*A*_^*S*^ value calculated separately for lineage A was also significantly different from zero (*I*_*A*_^*S*^ = 0.3263, *P* < 0.001). However, even in case of lineage B, which was found to undergo a high recombination rate, an *I*_*A*_^*S*^ value of 0.7171 (*P* < 0.001) indicated persistence of clonally evolving eSTs over time (Table 3).

## Discussion

To our knowledge, this is the first study that explored the phylodistribution of *M. hominis* clinical strains involved in distinct pathological conditions. For this purpose, we developed and assessed an eMLST scheme, combining both MLST and MVLST gene loci. While the set of MLST loci was previously reported^20^, the MVLST gene sequences were included for the first time, and have been selected based on their polymorphic nature, as previously demonstrated^23–26^.

The majority of strains, collected over a relatively long period of 17 years, were isolated from infertile patients (83%). This finding is in line with previous studies, which revealed the higher incidence of *M. hominis* in the genitourinary tract of both women and men presenting with infertility^27,28^.

The ten gene loci targeted in our eMLST scheme were all readily amplified by PCR, making them good candidates for multilocus sequence typing of *M. hominis*^29^. Despite the relatively small number of isolates included in this study (59 strains), 21 STs, 29 VTs, and 30 eST were distinguished by MLST, MVLST, and eMLST, respectively. Such a finding lends further support to the heterogeneous nature of *M. hominis*, as reported previously^14,21^. Hence, it seems clearly that increased genetic diversity of *M. hominis* was mainly brought about by virulence genes, as indicated by their average of haplotype diversity of 0.83. This might be the result of their frequent exposure to environmental changes. By contrast, and as expected, the average of haplotype diversity of 0.61 found in *M. hominis* housekeeping genes was comparable to other mycoplasmas species, such as *Mycoplasma bovis* (average of 0.64)^17^ and *Mycoplasma hyorhinis* (average of 0.651)^30^. Previous studies have confirmed that virulence gene-based typing schemes (MVLST) afford higher resolution than did MLST, making them the best surrogate approaches to carry out reliable epidemiological investigations^31,32^. However, neither MVLST nor MLST data could be unambiguously correlated with the clinical status of the studied strain collections.

Here, owing to the development of the eMLST scheme, we have been able to unambiguously elucidate the genetic relatedness of 59 *M. hominis* strains and their clear categorization into two distinct lineages, with each corresponding to a specific pathotype. Indeed, all infertility-associated strains fell into one lineage (lineage A), while all gynecological infections-associated strains belonged to another separate lineage (lineage B). Such a clear distinction between the two pathotypes could not be established either by MLST or MVLST gene sets-derived data. This finding was consistent with the conclusions drawn from previous studies, showing that the combination of housekeping and virulence genes provides a more convenient tool for studying the epidemiology of pathogenic bacteria. For example, the combination of MLST and MVLST analyses of *Vibrio cholerae* was able to differentiate toxigenic from non-toxigenic strains^31^. Moreover, a correlation between ureaplasma subgroup 2 and genitourinary tract disease outcomes has been revealed by an eMLST scheme^33^.

The segregation of *M. hominis* pathotypes into two distinct genetic lineages may help to identify pathogenicity determinants which might locate to lineage-specific genetic changes. However, the eMLST scheme developed herein should be tested on larger, clinically and geographically diverse, strain collections to confirm its promising value.

Our data indicate that despite an overall clonal evolution of *M. hominis* population, recombination could play a role in generating diversity, as previously suggested^20^. This was particularly noticeable within lineage B (gynecological infections-associated pathotype), since recombination was found to occur more frequently than mutation, a finding in sharp contrast with the mutation-driven evolution of lineage A (infertitlity-associated pathotype). However, both lineages displayed *I*^*S*^_*A*_ values indicative of significant linkage disequilibrium between their respective eSTs suggesting a clonal population structure. The observed recombination within lineage B is irreconcilable with a strictly clonal population structure. Theoretically, a clonal population structure under a high rate of recombination can be observed due to non-random and limited sampling, as in this case. Furthermore, estimation of linkage disequilibrium using a single representative from each of the 5 eSTs indicated slightly lower but still significant non-zero *I*^*S*^_*A*_ values, excluding the hypothesis of an epidemic population structure^34^. The clonal population structure of *M. hominis* is also supported by the observation that the same genotypes are repeatedly isolated over the years. For example, eST3, which is the most frequent genotype, was represented by isolates from temporally separated periods (2004–2016), suggesting that this type may be more fit than other potential variants. A recent study has also highlighted the existence of *Ureaplasma urealyticum* specific epidemic clonal lineages that are more likely to be transmitted between infertile couples and to be associated with clinical manifestations^35^.

Yet, the fact that recombination did not disrupt the phylogeny of *M. hominis* could be explained by purifying selection which retrieved deleterious amino acid changes in order to preserve the functionally optimal gene sequence. Indeed, both housekeeping and virulence genes were found to be evolving under purifying selection as suggested by *d*_*N*_/*d*_*S*_ ratios and the negative values of Tajima’s *D* statistic. This finding is not without precedent, since similar results have been reported for *Listeria monocytogenes*^36^ and *Staphylococcus lugdunensis*^32^. Hence, aside from preserving the most fit pathotype clones, purifying selection might also contribute to maintain gene sequences that define a given pathotype.

The main weakness of this study consists in the limited size of the sample set and the lack of geographical and clinical diversity. However, despite these limitations, the majority of our results (heterogeneity of *M. hominis*, occurrence of recombination, evolution of *M. hominis* under purifying selection, and the ability of eMLST to differentiate distinct genetic lineages) was in sharp concordance with previous reports^12,20,23,31,33^.

## Conclusion

This study demonstrated the usefulness of an eMLST scheme as a phylogenetic tool that could categorize the pathotypic nature of *M. hominis* clinical strains. Inclusion of a set of putative virulence genes endowed the eMLST typing scheme with increased discriminatory power and biological significance. Furthermore, our study provided insights into the selective forces driving *M. hominis* evolution. The overall picture that emerged from the obtained data shows that *M. hominis* undergoes genetic diversification through the combined action of mutation and recombination, while preserving the pathotype distribution, and/or their most fit clones, by evolving under purifying selection. These findings should prompt additional validation studies using larger, geographically diverse and clinically well defined, strain collections in order to thoroughly address the genetic relationship among distinct *M. hominis* pathotypes.

## Material and Methods

### Ethical clearance

No interventions were performed in this study. Only fully anonymized data were processed, and hence no further ethical clearance was required.

### Bacterial isolates

A total of 59 *M. hominis* isolates was collected between the years 2000 and 2017, and had been identified as previously described^23^. Of these, 49 isolates (83%) were recovered from infertile patients and 10 strains (17%) from patients suffering from gynecological infections (Supplementary Table S2). *M. hominis* reference strain PG21 (ATCC 23114) was included in this study.

### DNA extraction

For PCR, samples were prepared as described by kong *et* *al*.^37^. Briefly, cells from 2 ml of logarithmic-phase culture of each *M. hominis* isolate were harvested by centrifugation at 24,000 × g for 20 min. DNA was isolated by treatment with 200 μl of digestion buffer (10 mM Tris-HCl, pH 8.0; 0.45% Tween 20) and proteinase K (100 μg/ml). Lysates were incubated for 1 h at 60 °C, then for 10 min at 100 °C, and finally stored at −20 °C.

### Development of eMLST for *M. hominis* typing

An eMLST scheme consisting in the combination of housekeeping and putative virulence gene loci was developed. The set of housekeeping genes (*gyrB*, *tuf*, *ftsY*, *uvrA*, *gap)* was previously published and used as an MLST scheme^20^. Here we first screened 8 known *M. hominis* putative virulence genes, *p120*, *p120’*, *lmp1*, *lmp3*, *vaa*, *p60*, *p75*, and *p80*, in order to be included in our eMLST scheme, as a multi-virulence-locus sequence typing (MVLST) gene subset. To be included in the MVLST gene subset, a gene must be (i) amplified in all strains, (ii) amplified and sequenced using a single primer pair, and (iii) endowed with a higher discriminatory power. Only five virulence genes (*p120’*, *vaa*, *lmp1*, *lmp3*, *p60*) met the above inclusion criteria. Hence, our eMLST scheme consisted of a 10-loci combination (*gyrB*, *tuf*, *ftsY*, *uvrA*, *gap, p120’*, *vaa*, *lmp1*, *lmp3*, *p60*).

### PCR amplification and DNA sequencing

The primer sequences of each selected locus are displayed in Supplementary Table S3. All amplifications were performed in a final volume of 50 µl consisting of 1X PCR buffer (10 mM Tris-HCl, pH 8.3; 50 mM KCl) (Invitrogen, USA), 2.5 mM MgCl_2_ (Invitrogen, USA), 0.4 µM of each primer (Sigma-Aldrich, Germany), 200 µM dNTPs (Sigma-Aldrich, Germany), 1.25 U of *Taq* DNA polymerase (Invitrogen, USA), and 10 µl of treated sample. An Applied Biosystems Thermal Cycler 2720 (Life Technologies, USA) was set up with a first cycle of denaturation for 5 min at 94 °C, followed by 35 cycles of denaturation at 94 °C for 0.45 min, annealing at 58 °C for 0.45 min, elongation at 72 °C for 1 min, and a final extension step of 7 min at 72 °C. The PCR products were electrophoresed on 1.5% agarose gel and stained with ethidium bromide (Sigma-Aldrich, Germany) to visualize the DNA bands, whose sizes were determined using a 100-bp DNA ladder (Invitrogen, USA). PCR products were then purified with both Exonuclease I (Biolabs, England) and Shrimp Alkaline Phosphatase (Biolabs, England) as per manufacturer’s instructions. Sequencing reactions were performed using a BigDye Terminator v3.1 cycle sequencing kit (Applied Biosystems, USA), according to the manufacturer’s recommendations, and were run in an ABI 3130 genetic analyzer (Applied Biosystems, USA). Both strands from each PCR amplicon were sequenced twice.

### Descriptive analysis of MLST, MVLST, and eMLST data

The nucleotide sequences obtained were aligned with reference sequences downloaded from GenBank by using BioEdit Sequence Alignment Editor version 7.2.5^38^. For each locus, distinct allele sequences were assigned an arbitrary allele number for identification. Each isolate was characterized by a pattern of numbers defining its allelic profile: sequence type (ST) for MLST (*uvrA*, *gyrB*, *ftsY*, *tuf*, *gap*) and virulence type (VT) for MVLST (*p120’*, *vaa*, *lmp1*, *lmp3*, *p60*) gene sets. An expanded sequence type (eST) was assigned for the 10 eMLST genes of all tested strains.

### Diversity analysis of nucleotide sequences

Information for each locus including the number of polymorphic sites and the haplotype diversity (H) were calculated using DnaSP genetic software version 5.1^39^. To investigate whether positive or negative selection had occurred at the protein level, the average non-synonymous/synonymous substitution rate ratio (*d*_*N*_/*d*_*S*_) was calculated. *d*_*N*_/*d*_*S*_ < 1 indicates that the relative genes were mainly affected by purifying selection during the population evolution, *d*_*N*_/*d*_*S*_ = 1 indicates neutral selection, and *d*_*N*_/*d*_*S*_ > 1 indicates positive selection. The DnaSP software was also used to calculate Tajima’s *D* statistic in order to test the neutrality of the observed DNA polymorphisms^40^.

### Evaluation of eMLST

The discriminatory power, the degree of congruence among different typing schemes, and the statistical analysis were determined via an online tool (http://www.comparingpartitions.info/). The discriminatory ability was evaluated using the Simpson’s index of diversity, SID, with 95% confidence intervals, as described by Hunter and Gaston^41^. It refers to the probability that two strains sampled randomly in the collection belong to two different types. An index greater than 0.90 is considered desirable if the typing results are to be interpreted with confidence^41^. Concordance between eMLST and other typing schemes (MLST or MVLST) was calculated using the Adjusted Rand index (AR)^42^ and the Adjusted Wallace coefficient (AW)^43^. AR value close to 0 indicates a lack of congruence between the typing schemes, while value close to 1 indicates a high level of congruence. AW value indicates the probability that two strains classified as the same type by one typing scheme will also be classified as the same one when using the other typing scheme.

### Population structure and phylogenetic analysis

To determine closely related genotypes within highly similar clusters, the data was analysed using the BURST version 3 algorithm implemented in the eBURST web server^44^ (http://eburst.mlst.net/). The software determined clonal complexes (CC) from the data, suggesting a consensus ancestral type. To analyse the 59 *M. hominis* strains, we applied a DLV (Double Locus Variable) criteria for all schemes. This means that the maximum difference allowed within the same CC was that of two loci.

Three phylogenetic trees from the concatenated sequences of MLST (2361 pb), MVLST (1728 pb), and eMLST (4089 bp) gene sets, and phylogenetic trees based on the allele sequences of each gene, were constructed by the Neighbor-Joining (NJ)^45^ method with a Kimura’s two-parameter distance model and 1,000 bootstrap replications using MEGA version 6.06^46^. STRUCTURE software version 2.3.4^47,48^ with linkage model was used to identify the ancestral populations for each eST. The *K-*value that generated the highest posterior probability was used as the probable number of ancestral populations. Ten individual runs per value of *K* (chosen between 2 and 10) were performed using 200,000 burn-in iterations and 300,000 Markov Chain Monte Carlo (MCMC) iterations. The STRUCTURE Harvester^49^, which implements the Evanno method, was used to identify the most probable groups (*K*) that best fit the data. The *K*-value that generated the highest median posterior probability was used as the probable number of ancestral populations. We estimated the fixation index (*F*_ST_) to mesure the degree of population differenciation using 100,000 genotypic permutations in Arlequin 3.5^50^. In general, *F*_ST_ values range from 0 (no differentiation) to 1 (complete differentiation). Basically, an *F*_ST_ value of 0.05–0.15 indicates low differentiation, while *F*_ST_ > 0.30 indicates high differentiation^51^.

The relationships between the eST and the clinical manifestations of all isolates were determined using a Minimum Spanning Tree (MST) analysis performed with BioNumerics software (version 7.0, Applied Maths).

### Recombination analysis

The split network of eSTs and individual loci were generated by using neighbor-net method using SplitTree4^52^. The pairwise homoplasy index (*phi*) test^53^ implemented in SplitTree4 for recombination was performed, and the *P*-value < 0.05 indicated recombination existed. LDhat programme implemented in Recombination Detection Program (RDP) version 4.83^54^ was used to calculate the per site recombination/mutation ratio (ρ/θ)^55^ based on the concatenated sequences of ten loci with 100,000 MCMC iterations. Linkage disequilibrium from allelic data was evaluated by calculating the standardized index of association (*I*^*S*^_*A*_) using LIAN version 3.7^56,57^ (http://guanine.evolbio.mpg.de/cgi-bin/lian/lian.cgi.pl/query). *I*_*A*_^*S*^ value significantly different from 0 indicates that a population is clonal (linkage disequilibrium), while *I*_*A*_^*S*^ equal to zero indicates a recombining population structure (linkage equilibrium). *I*_*A*_^*S*^ was tested by using Monte Carlo methods with 1,000 iterations on allelic profile.

### Nucleotide sequence accession numbers

Nucleotide sequences of the ten eMLST allelic variants were submitted to GenBank, and their accession numbers are provided in Supplementary Table S4.

## Electronic supplementary material

Supplementary Information

## Data Availability

All data generated and analysed during this study are included in this published article and its Supplementary Information file.
