# Supplementary material for: Phylogenetics of Mycoplasma hominis clinical strains associated with gynecological infections or infertility as disclosed by an expanded multilocus sequence typing scheme
Source: Sci Rep. 2018 Oct 5;8:14854. doi: 10.1038/s41598-018-33260-x (PMC6173709; doi:10.1038/s41598-018-33260-x)
Supplement: Supplementary file 1 — Supplementary Information [file 41598_2018_33260_MOESM1_ESM.doc]

**Phylogenetics of *Mycoplasma hominis* clinical strains associated with gynecological infections or infertility as** **disclosed by an expanded multilocus sequence typing scheme**

**Safa Boujemaa^1^, Amina Ben Allaya^1^, Béhija Mlik^1^, Helmi Mardassi^2^ & Boutheina Ben Abdelmoumen Mardassi^1^*.**

^1^Group of Mycoplasmas, Laboratory of Molecular Microbiology, Vaccinology, and Biotechnology Development.

^2^Unit of Typing & Genetics of Mycobacteria, Laboratory of Molecular Microbiology, Vaccinology, and Biotechnology Development.

Institut Pasteur de Tunis, Université de Tunis El Manar, 13, Place Pasteur, BP 74, 1002, Tunis Belvédère, Tunisia.

*Corresponding author: Tel: +216 71 847 609

E-mail address: [boutheina.mardassi@pasteur.tn](mailto:boutheina.mardassi@pasteur.tn)

| **Strain name** | **eMLST** | | | | | | | | | | | | |
| --- | --- | --- | --- | --- | --- | --- | --- | --- | --- | --- | --- | --- | --- |
|  | **MLST** | | | | | | **MVLST** | | | | | | **eST** |
|  | ***uvrA*** | ***gyrB*** | ***ftsY*** | ***tuf*** | ***gap*** | **ST** | ***p120'*** | ***vaa*** | ***lmp1*** | ***lmp3*** | ***p60*** | **VT** |  |
| PG21 | 1 | 1 | 1 | 1 | 1 | 1 | 1 | 1 | 1 | 1 | 1 | 1 | 1 |
| MH1 | 1 | 1 | 1 | 1 | 1 | 1 | 1 | 1 | 1 | 1 | 1 | 1 | 1 |
| MH2 | 2 | 1 | 1 | 2 | 2 | 9 | 8 | 3 | 8 | 7 | 6 | 9 | 9 |
| MH3 | 2 | 1 | 1 | 7 | 2 | 10 | 8 | 5 | 11 | 7 | 6 | 10 | 10 |
| MH4 | 4 | 1 | 1 | 3 | 3 | 2 | 2 | 2 | 3 | 3 | 2 | 2 | 2 |
| MH5 | 4 | 1 | 1 | 3 | 3 | 2 | 2 | 2 | 3 | 3 | 2 | 2 | 2 |
| MH6 | 4 | 1 | 1 | 3 | 3 | 2 | 2 | 2 | 3 | 3 | 2 | 2 | 2 |
| MH7 | 4 | 1 | 1 | 3 | 3 | 2 | 2 | 2 | 3 | 3 | 2 | 2 | 2 |
| MH8 | 4 | 1 | 1 | 2 | 3 | 4 | 2 | 2 | 2 | 3 | 2 | 11 | 11 |
| MH9 | 4 | 1 | 1 | 3 | 3 | 2 | 2 | 2 | 3 | 3 | 2 | 2 | 2 |
| MH10 | 2 | 1 | 1 | 2 | 3 | 6 | 4 | 1 | 2 | 3 | 2 | 12 | 12 |
| MH11 | 7 | 1 | 1 | 6 | 9 | 11 | 9 | 4 | 9 | 9 | 7 | 14 | 14 |
| MH12 | 2 | 2 | 3 | 5 | 6 | 8 | 3 | 1 | 4 | 4 | 2 | 8 | 8 |
| MH13 | 4 | 1 | 1 | 2 | 3 | 4 | 2 | 2 | 2 | 2 | 2 | 4 | 4 |
| MH14 | 4 | 1 | 1 | 1 | 1 | 13 | 2 | 2 | 1 | 2 | 1 | 13 | 13 |
| MH15 | 2 | 1 | 1 | 2 | 2 | 9 | 3 | 1 | 4 | 4 | 9 | 15 | 15 |
| MH16 | 4 | 1 | 1 | 2 | 3 | 4 | 2 | 2 | 2 | 2 | 2 | 4 | 4 |
| MH17 | 1 | 1 | 1 | 1 | 1 | 1 | 1 | 1 | 1 | 1 | 1 | 1 | 1 |
| MH18 | 2 | 2 | 3 | 5 | 6 | 8 | 3 | 1 | 4 | 4 | 2 | 8 | 8 |
| MH19 | 2 | 1 | 1 | 11 | 9 | 12 | 10 | 3 | 7 | 8 | 6 | 24 | 24 |
| MH20 | 4 | 1 | 1 | 2 | 3 | 4 | 2 | 2 | 2 | 2 | 2 | 4 | 4 |
| MH21 | 4 | 1 | 1 | 2 | 3 | 4 | 2 | 2 | 2 | 2 | 2 | 4 | 4 |
| MH22 | 2 | 2 | 3 | 5 | 6 | 8 | 3 | 1 | 4 | 4 | 2 | 8 | 8 |
| MH23 | 7 | 1 | 1 | 2 | 10 | 14 | 11 | 4 | 5 | 2 | 7 | 17 | 17 |
| MH24 | 3 | 1 | 1 | 4 | 4 | 3 | 5 | 1 | 6 | 5 | 3 | 3 | 3 |
| MH25 | 1 | 1 | 1 | 1 | 1 | 1 | 2 | 1 | 1 | 1 | 1 | 18 | 18 |
| MH26 | 9 | 1 | 4 | 10 | 3 | 19 | 7 | 1 | 10 | 2 | 5 | 19 | 19 |
| MH27 | 6 | 3 | 5 | 8 | 5 | 16 | 6 | 2 | 5 | 6 | 1 | 7 | 7 |
| MH28 | 3 | 1 | 1 | 4 | 4 | 3 | 5 | 1 | 6 | 5 | 3 | 3 | 3 |
| MH29 | 3 | 1 | 1 | 4 | 4 | 3 | 5 | 1 | 6 | 5 | 3 | 3 | 3 |
| MH30 | 2 | 1 | 1 | 2 | 3 | 6 | 4 | 1 | 2 | 2 | 2 | 5 | 5 |
| MH31 | 10 | 1 | 1 | 2 | 7 | 15 | 7 | 3 | 12 | 10 | 8 | 21 | 21 |
| MH32 | 1 | 1 | 1 | 1 | 1 | 1 | 1 | 1 | 1 | 1 | 1 | 1 | 1 |
| MH33 | 6 | 3 | 5 | 8 | 5 | 16 | 6 | 2 | 5 | 6 | 1 | 7 | 7 |
| MH34 | 2 | 1 | 1 | 2 | 3 | 6 | 4 | 1 | 2 | 2 | 2 | 5 | 5 |
| MH35 | 3 | 1 | 1 | 4 | 4 | 3 | 5 | 2 | 6 | 5 | 3 | 6 | 6 |
| MH36 | 11 | 1 | 1 | 4 | 4 | 17 | 2 | 3 | 6 | 5 | 3 | 23 | 23 |
| MH37 | 6 | 3 | 5 | 8 | 5 | 16 | 2 | 2 | 5 | 6 | 1 | 16 | 16 |
| MH38 | 3 | 1 | 5 | 9 | 3 | 18 | 12 | 2 | 13 | 5 | 3 | 25 | 25 |
| MH39 | 3 | 1 | 1 | 4 | 4 | 3 | 5 | 2 | 6 | 5 | 3 | 6 | 6 |
| MH40 | 3 | 1 | 6 | 6 | 8 | 20 | 2 | 1 | 15 | 11 | 10 | 26 | 26 |
| MH41 | 2 | 1 | 1 | 2 | 3 | 6 | 4 | 1 | 2 | 2 | 2 | 5 | 5 |
| MH42 | 3 | 1 | 1 | 4 | 4 | 3 | 5 | 1 | 6 | 5 | 3 | 3 | 3 |
| MH43 | 3 | 1 | 1 | 4 | 4 | 3 | 5 | 1 | 6 | 5 | 3 | 3 | 3 |
| MH44 | 6 | 3 | 5 | 8 | 5 | 16 | 6 | 2 | 5 | 6 | 1 | 7 | 7 |
| MH45 | 3 | 1 | 1 | 4 | 4 | 3 | 5 | 1 | 6 | 5 | 3 | 3 | 3 |
| MH46 | 3 | 1 | 1 | 4 | 4 | 3 | 5 | 1 | 6 | 5 | 3 | 3 | 3 |
| MH47 | 3 | 1 | 1 | 4 | 4 | 3 | 5 | 1 | 6 | 5 | 3 | 3 | 3 |
| MH48 | 3 | 1 | 1 | 4 | 4 | 3 | 5 | 1 | 6 | 5 | 3 | 3 | 3 |
| MH49 | 6 | 3 | 5 | 8 | 5 | 16 | 6 | 2 | 5 | 6 | 1 | 7 | 7 |
| MH50 | 5 | 1 | 2 | 6 | 6 | 5 | 1 | 3 | 14 | 11 | 4 | 20 | 20 |
| MH51 | 8 | 1 | 1 | 4 | 10 | 7 | 7 | 1 | 16 | 12 | 5 | 27 | 27 |
| MH52 | 5 | 1 | 2 | 6 | 6 | 5 | 1 | 3 | 14 | 11 | 4 | 20 | 20 |
| MH53 | 8 | 1 | 1 | 4 | 10 | 7 | 7 | 1 | 17 | 11 | 5 | 28 | 28 |
| MH54 | 4 | 1 | 1 | 3 | 3 | 2 | 2 | 2 | 3 | 2 | 2 | 22 | 29 |
| MH55 | 2 | 1 | 1 | 2 | 3 | 6 | 2 | 2 | 2 | 2 | 2 | 4 | 22 |
| MH56 | 2 | 1 | 1 | 4 | 10 | 21 | 13 | 6 | 18 | 13 | 1 | 29 | 30 |
| MH57 | 2 | 1 | 1 | 4 | 10 | 21 | 13 | 6 | 18 | 13 | 1 | 29 | 30 |
| MH58 | 2 | 1 | 1 | 4 | 10 | 21 | 13 | 6 | 18 | 13 | 1 | 29 | 30 |
| MH59 | 2 | 1 | 1 | 4 | 10 | 21 | 13 | 6 | 18 | 13 | 1 | 29 | 30 |
|  | | | | | | | | | | | | | |

**Supplementary Table S1. MLST, MVLST, and eMLST allelic profiles of *M. hominis* isolates.**

| **Strain Name** | **Year of isolation** | **Source** | **Sex** | **Clinical Manifestation** |
| --- | --- | --- | --- | --- |
|
|
| PG21 | 1953 | rectal swab | Unknown | Healthy individual |
| MH1 | 2000 | vaginal swab | Female | Gynecological infections |
| MH2 | 2006 | semen | Male | Infertility |
| MH3 | 2010 | vaginal swab | Female | Infertility |
| MH4 | 2005 | vaginal swab | Female | Infertility |
| MH5 | 2003 | vaginal swab | Female | Infertility |
| MH6 | 2002 | vaginal swab | Female | Infertility |
| MH7 | 2001 | vaginal swab | Female | Infertility |
| MH8 | 2000 | semen | Male | Infertility |
| MH9 | 2010 | vaginal swab | Female | Infertility |
| MH10 | 2008 | semen | Male | Infertility |
| MH11 | 2007 | semen | Male | Infertility |
| MH12 | 2005 | semen | Male | Infertility |
| MH13 | 2005 | vaginal swab | Female | Infertility |
| MH14 | 2003 | semen | Male | Gynecological infections |
| MH15 | 2012 | vaginal swab | Female | Infertility |
| MH16 | 2005 | vaginal swab | Female | Infertility |
| MH17 | 2010 | vaginal swab | Female | Gynecological infections |
| MH18 | 2006 | semen | Male | Infertility |
| MH19 | 2011 | semen | Male | Infertility |
| MH20 | 2007 | vaginal swab | Female | Infertility |
| MH21 | 2014 | semen | Male | Infertility |
| MH22 | 2010 | vaginal swab | Female | Infertility |
| MH23 | 2012 | semen | Male | Infertility |
| MH24 | 2004 | vaginal swab | Female | Infertility |
| MH25 | 2015 | vaginal swab | Female | Gynecological infections |
| MH26 | 2014 | vaginal swab | Female | Infertility |
| MH27 | 2009 | vaginal swab | Female | Gynecological infections |
| MH28 | 2006 | vaginal swab | Female | Infertility |
| MH29 | 2010 | vaginal swab | Female | Infertility |
| MH30 | 2008 | vaginal swab | Female | Infertility |
| MH31 | 2015 | vaginal swab | Female | Infertility |
| MH32 | 2010 | vaginal swab | Female | Gynecological infections |
| MH33 | 2007 | vaginal swab | Female | Gynecological infections |
| MH34 | 2003 | vaginal swab | Female | Infertility |
| MH35 | 2008 | vaginal swab | Female | Infertility |
| MH36 | 2012 | vaginal swab | Female | Infertility |
| MH37 | 2011 | vaginal swab | Female | Gynecological infections |
| MH38 | 2005 | vaginal swab | Female | Infertility |
| MH39 | 2004 | semen | Male | Infertility |
| MH40 | 2013 | vaginal swab | Female | Infertility |
| MH41 | 2012 | vaginal swab | Female | Infertility |
| MH42 | 2005 | semen | Male | Infertility |
| MH43 | 2007 | vaginal swab | Female | Infertility |
| MH44 | 2011 | semen | Male | Gynecological infections |
| MH45 | 2016 | vaginal swab | Female | Infertility |
| MH46 | 2013 | vaginal swab | Female | Infertility |
| MH47 | 2011 | vaginal swab | Female | Infertility |
| MH48 | 2009 | vaginal swab | Female | Infertility |
| MH49 | 2011 | vaginal swab | Female | Gynecological infections |
| MH50 | 2011 | semen | Male | Infertility |
| MH51 | 2015 | vaginal swab | Female | Infertility |
| MH52 | 2011 | vaginal swab | Female | Infertility |
| MH53 | 2010 | vaginal swab | Female | Infertility |
| MH54 | 2009 | vaginal swab | Female | Infertility |
| MH55 | 2016 | semen | Male | Infertility |
| MH56 | 2017 | semen | Male | Infertility |
| MH57 | 2017 | vaginal swab | Female | Infertility |
| MH58 | 2017 | semen | Male | Infertility |
| MH59 | 2017 | vaginal swab | Female | Infertility |

**Supplementary Table S2. Epidemiologic characteristics of *M. hominis* strains used in this study.**

| **Target gene** | **Primer** | **Sequence (5’ to 3’)** | **GenBank accession number** | **Reference** |
| --- | --- | --- | --- | --- |
| *uvrA* | uvraf  uvrar | GTACATGTTAATCCCAGAAGTATAGTTGG  GCATTCAGGACACATTCCGG | CAX37477.1 | 20 |
| *gyrB* | gyrbf  gyrbr | GGATTACATGGTGGTGGTGC  TCCTTCTGCATAAATAACTTCGGG | [CAX37511](http://www.ebi.ac.uk/ena/data/view/CAX37511).1 | 20 |
| *ftsY* | ftsyf  ftsyr | GGTACCAAAGAAGAAAGAATAGCC  TCCTGAACCATTGACACCC | CAX37594.1 | 20 |
| *tuf* | tuff  tufr | GCTGCTACTGATGGTCCTATGC  ACCAGTTGCAACAGTTCCACG | CAX37186.1 | 20 |
| *gap* | P33  P42 | GGTTTTGGTGGAATTGGTCG  TGCTCTTCTTAAATCTTTGTGTGGAGC | CAX37649.1 | 20 |
| *p120'* | Mhp120'f  Mhp120'r | TGAGGAATTTCAACTGGTGTCC  TGTTGTAATAGCATTTAAG | CAX37515.1 | 23 |
| *vaa* | MhvaaPf  MhvaaPr | GGAATAGCAACTACTGCTATTTTGC  TTGATCAACTTTTTGTTGTTCACTTTTTGCG | CAX37482.1 | This study |
| *lmp1* | MhLmp1f  MhLmp1r | ACTCCAATTAGTCTTGAATC  TGAGGCAACTGCTTGATTTGC | CAX37187.1 | This study |
| *lmp3* | MhLmp3f  MhLmp3r | GACGGTCAAAACGTCGATTC  AAGTTCTTGTTGTTTGG | CAX37298.1 | This study |
| *p60* | p60f  p60r | GTTACAAAAGACAATTCAA  TACTAGACCTTCAACTTTC | CAX37484.1 | This study |

**Supplementary Table S3. Genes and respective flanking oligonucleotide primers used in the *Mycoplasma hominis* eMLST scheme.**

| **Gene** | **Allele** | **Accession number** |
| --- | --- | --- |
| *uvrA* | 1 | MG879464 |
|  | 2 | MG879465 |
|  | 3 | MG879466 |
|  | 4 | MG879467 |
|  | 5 | MG879468 |
|  | 6 | MG879469 |
|  | 7 | MG879470 |
|  | 8 | MG879471 |
|  | 9 | MG879472 |
|  | 10 | MG879473 |
|  | 11 | MG879474 |
| *gyrB* | 1 | MG879450 |
|  | 2 | MG879451 |
|  | 3 | MG879452 |
| *ftsY* | 1 | MG879434 |
|  | 2 | MG879435 |
|  | 3 | MG879436 |
|  | 4 | MG879437 |
|  | 5 | MG879438 |
|  | 6 | MG879439 |
| *tuf* | 1 | MG879453 |
|  | 2 | MG879454 |
|  | 3 | MG879455 |
|  | 4 | MG879456 |
|  | 5 | MG879457 |
|  | 6 | MG879458 |
|  | 7 | MG879459 |
|  | 8 | MG879460 |
|  | 9 | MG879461 |
|  | 10 | MG879462 |
|  | 11 | MG879463 |
| *gap* | 1 | MG879440 |
|  | 2 | MG879441 |
|  | 3 | MG879442 |
|  | 4 | MG879443 |
|  | 5 | MG879444 |
|  | 6 | MG879445 |
|  | 7 | MG879446 |
|  | 8 | MG879447 |
|  | 9 | MG879448 |
|  | 10 | MG879449 |
| *p120’* | 1 | MG879415 |
|  | 2 | MG879416 |
|  | 3 | MG879417 |
|  | 4 | MG879418 |
|  | 5 | MG879419 |
|  | 6 | MG879420 |
|  | 7 | MG879421 |
|  | 8 | MG879422 |
|  | 9 | MG879423 |
|  | 10 | MG879424 |
|  | 11 | MG879425 |
|  | 12 | MG879426 |
|  | 13 | MG879427 |
| *vaa* | 1 | MG879428 |
|  | 2 | MG879429 |
|  | 3 | MG879430 |
|  | 4 | MG879431 |
|  | 5 | MG879432 |
|  | 6 | MG879433 |
| *lmp1* | 1 | MG879374 |
|  | 2 | MG879375 |
|  | 3 | MG879376 |
|  | 4 | MG879377 |
|  | 5 | MG879378 |
|  | 6 | MG879379 |
|  | 7 | MG879380 |
|  | 8 | MG879381 |
|  | 9 | MG879382 |
|  | 10 | MG879383 |
|  | 11 | MG879384 |
|  | 12 | MG879385 |
|  | 13 | MG879386 |
|  | 14 | MG879387 |
|  | 15 | MG879388 |
|  | 16 | MG879389 |
|  | 17 | MG879390 |
|  | 18 | MG879391 |
| *lmp3* | 1 | MG879392 |
|  | 2 | MG879393 |
|  | 3 | MG879394 |
|  | 4 | MG879395 |
|  | 5 | MG879396 |
|  | 6 | MG879397 |
|  | 7 | MG879398 |
|  | 8 | MG879399 |
|  | 9 | MG879400 |
|  | 10 | MG879401 |
|  | 11 | MG879402 |
|  | 12 | MG879403 |
|  | 13 | MG879404 |
| *p60* | 1 | MG879405 |
|  | 2 | MG879406 |
|  | 3 | MG879407 |
|  | 4 | MG879408 |
|  | 5 | MG879409 |
|  | 6 | MG879410 |
|  | 7 | MG879411 |
|  | 8 | MG879412 |
|  | 9 | MG879413 |
|  | 10 | MG879414 |

**Supplementary Table S4. eMLST allelic profiles and their respective accession numbers.**


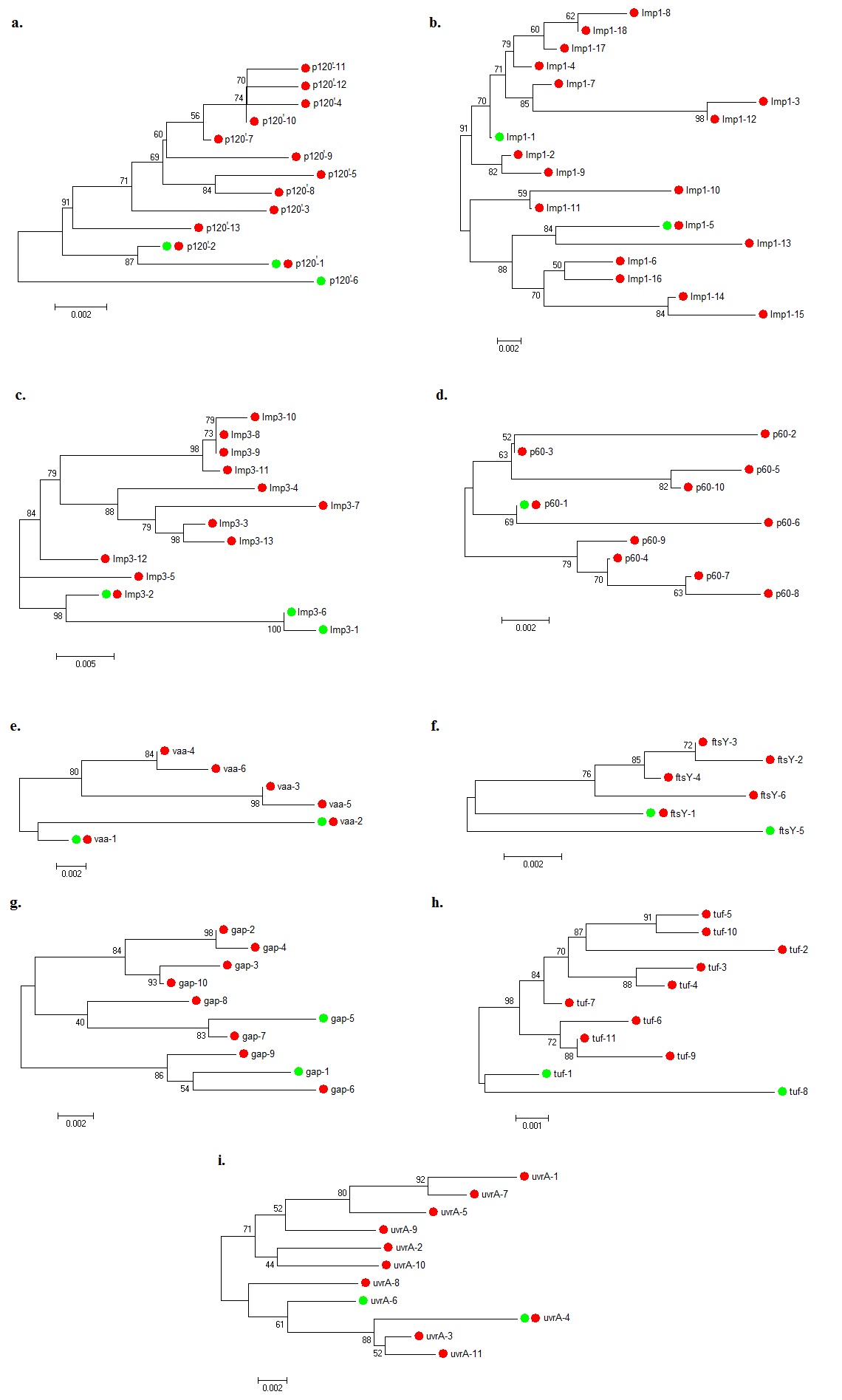


**Supplementary Figure S1. Neighbor-joining trees based on the allele sequences of *p120’* (a), *lmp1* (b), *lmp3* (c), *p60* (d), *vaa* (e), *ftsY* (f), *gap* (g), *tuf* (h), and *uvrA* (i) genes. The numbering in the figure refers to allele number. Bootstrap values (1,000 replications) are shown at the interior branches. Red circle refers to infertility and green circle refers to gynecological infections.**


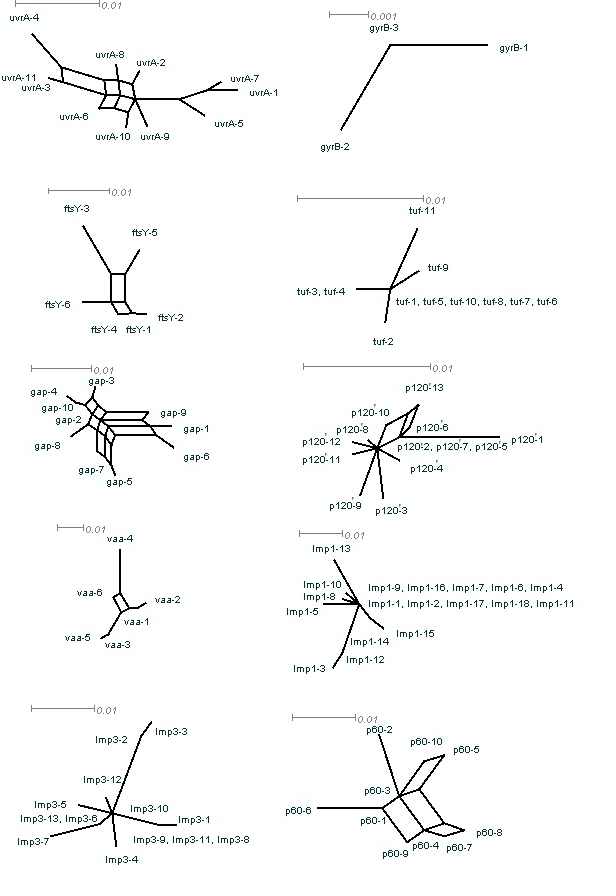


**Supplementary Figure S2. Split network analysis of ten individual eMLST loci. Multi-parallelogram formations indicate recombination events. The numbering in the figure refers to allele numbers.**
